# Experimental data on permeability-related properties of concrete containing nano natural pozzolana as cement replacement

**DOI:** 10.1016/j.dib.2022.108391

**Published:** 2022-06-17

**Authors:** Aref M. al-Swaidani

**Affiliations:** Faculty of Architectural Engineering, Arab International (Formerly European) University, Damascus, Syria

**Keywords:** Water permeability, Chloride ion penetrability, nano additives, natural pozzolana

## Abstract

The addition of supplementary cementitious materials (SCM), particularly at nano level, leads to several benefits, such as the reduced permeability of concrete matrix, which consequently leads to having more durable and sustainable concrete. The dataset presented in the current paper was collected from two series of experiments. The natural pozzolana used in making concrete mixtures is of volcanic origin; i.e. volcanic scoria. The first series was conducted to test nano natural pozzolana-based concrete specimens on water penetration, while the second one was carried out to investigate the chloride ion penetrability of the latter specimens. Concrete specimens were prepared using nano natural pozzolana of two sizes and six replacement levels. Concrete mixes of four water/binder ratios were prepared. All experiments were conducted after five curing times; namely 2, 7, 28, 90 and 180 days. The collected dataset would be beneficial for concrete mix designers, concrete properties analyzers, construction project managers and scientific researchers. An attempt to correlate water permeability with chloride ion penetrability was made.

## Specifications Table

**Table:** 

Subject	Civil and structural engineering, nanotechnology, materials science (general).
Specific subject area	Supplementary Cementitious Materials (SCM).
Type of data	Table Image Figure Excel sheets.
How the data were acquired	Water and chloride ion permeability tests after five curing times. The test results were used to assess the durability indicator of nano natural pozzolana.
Data format	Raw
Description of data collection	Data was collected from experiments conducted on concrete specimens after five curing times, namely 2, 7, 28, 90 and 180 days. Concrete mixtures were prepared using four w/b ratios, namely 0.4, 0.5, 0.6 and 0.7, six nano natural pozzolana contents, namely, 0, 1, 2, 3, 4 and 5% as cement replacement, two median particle sizes of nano volcanic scoria, namely 100 & 500 nm and aggregate blend. Water penetration depth and chloride ion penetrability tests were carried out.
Data source location	Faculty of Architectural Engineering, Arab International (Formerly European) University, Damascus, Syria.
Data accessibility	All experimental data are presented in this paper. Repository name: Mendeley Data identification number (DOI): 10.17632/m9tsgpvrtm.1 Direct link to the dataset: https://data.mendeley.com/datasets/m9tsgpvrtm/1
Related research article	-A.M. al-Swaidani, W.T. Khwies, M. al-Baly, T. Lala, Development of MLR, ANN & FL to predict the efficiency factor and durability indicator of nano natural pozzolana as cement additive, Journal of Building Engineering, 52(2022) 104475. https://doi.org/10.1016/j.jobe.2022.104475.

## Value of the Data

• The presented experimental data highlights an important aspect of concrete properties; i.e. concrete permeability, particularly when natural pozzolana is added as cement replacement at nano scale.
• Such kind of data can be useful for regions of similar geology.
• The designers of concrete mixtures may benefit from this data, particularly when concrete is to be used in aggressive environments.
• The available data with other data may help the researchers who are interested in developing predictive models.
• The wide range of w/b ratios, the replacement levels and the curing times may provide significant data on the performance of natural pozzolana-based cement concrete.

## 1 Data Description

**Fig. 1:**
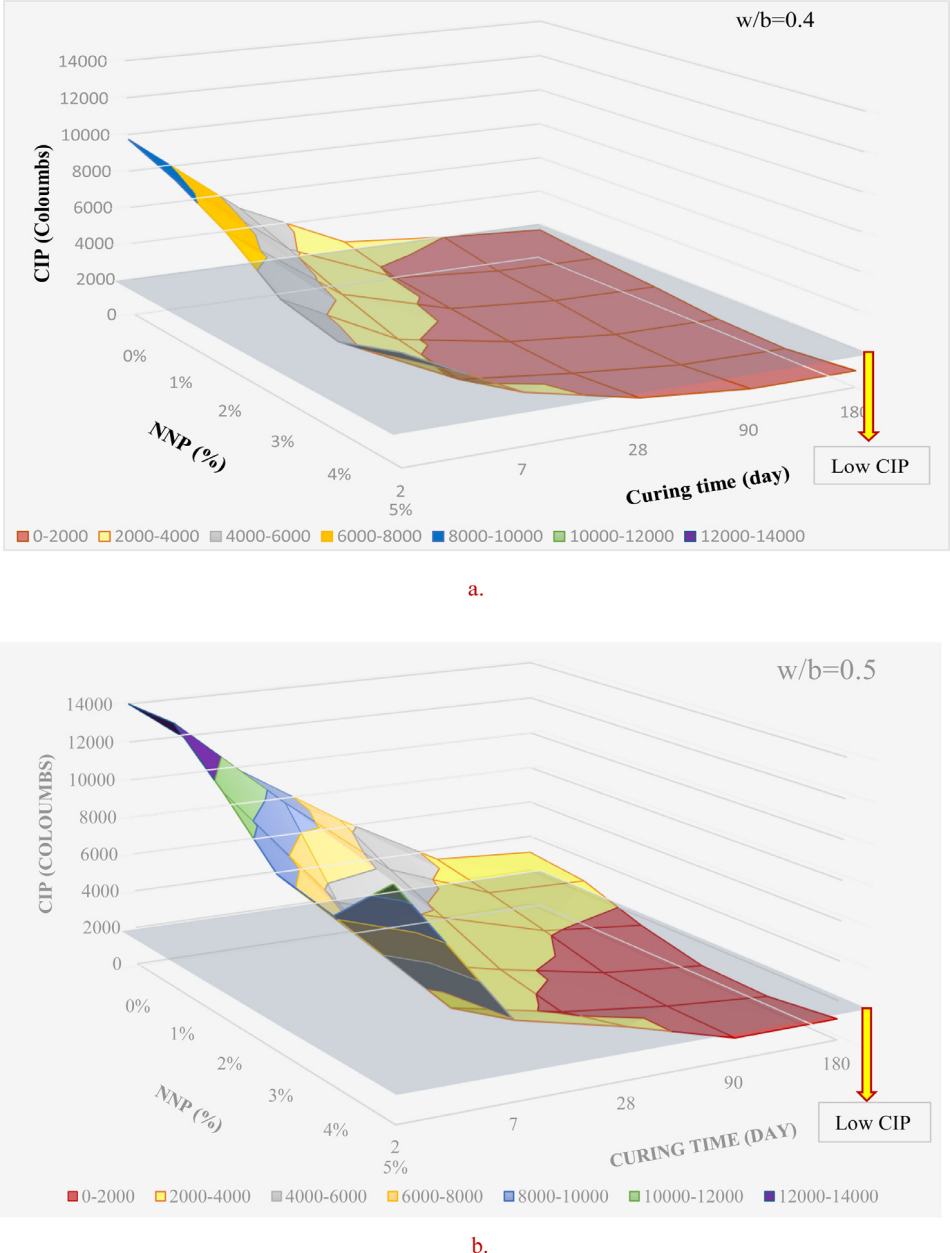

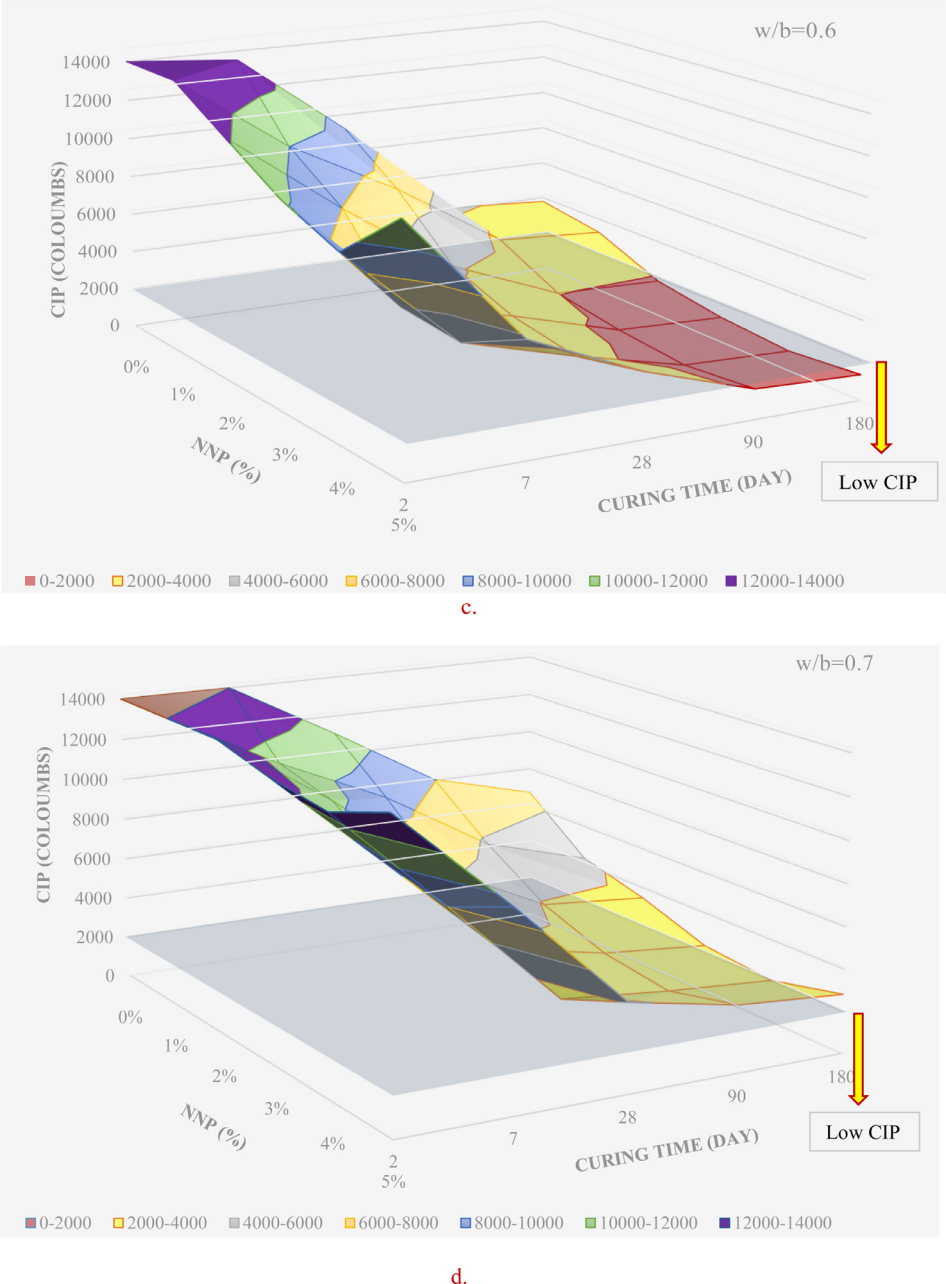
chloride ion penetrability (CIP) values of concrete mixtures prepared using nano natural pozzolana of 100 nm size with different w/b ratios.

**Fig. 2:**
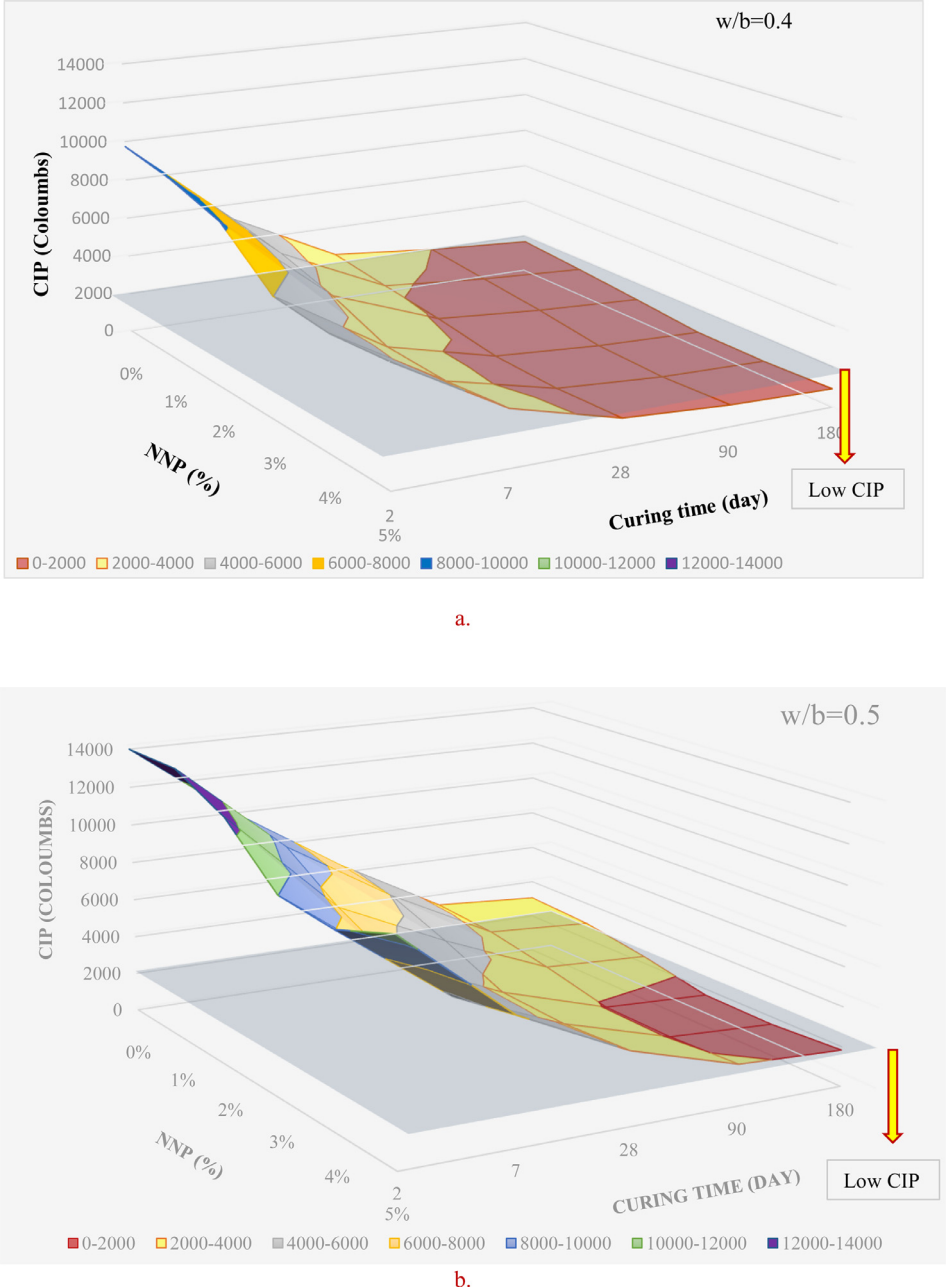

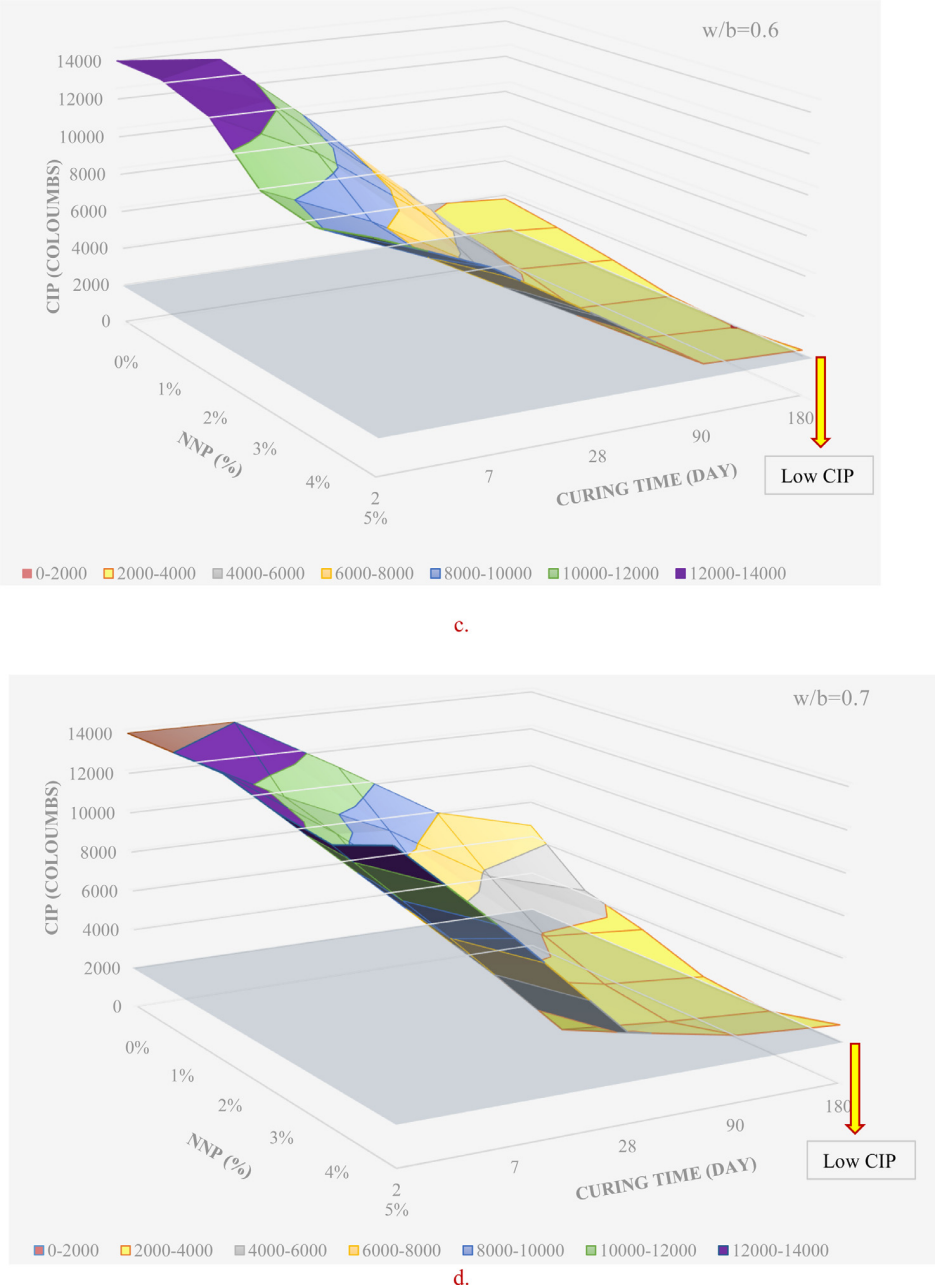
chloride ion penetrability (CIP) values of concrete mixtures prepared using nano natural pozzolana of 500 nm size.

**Fig. 3:**
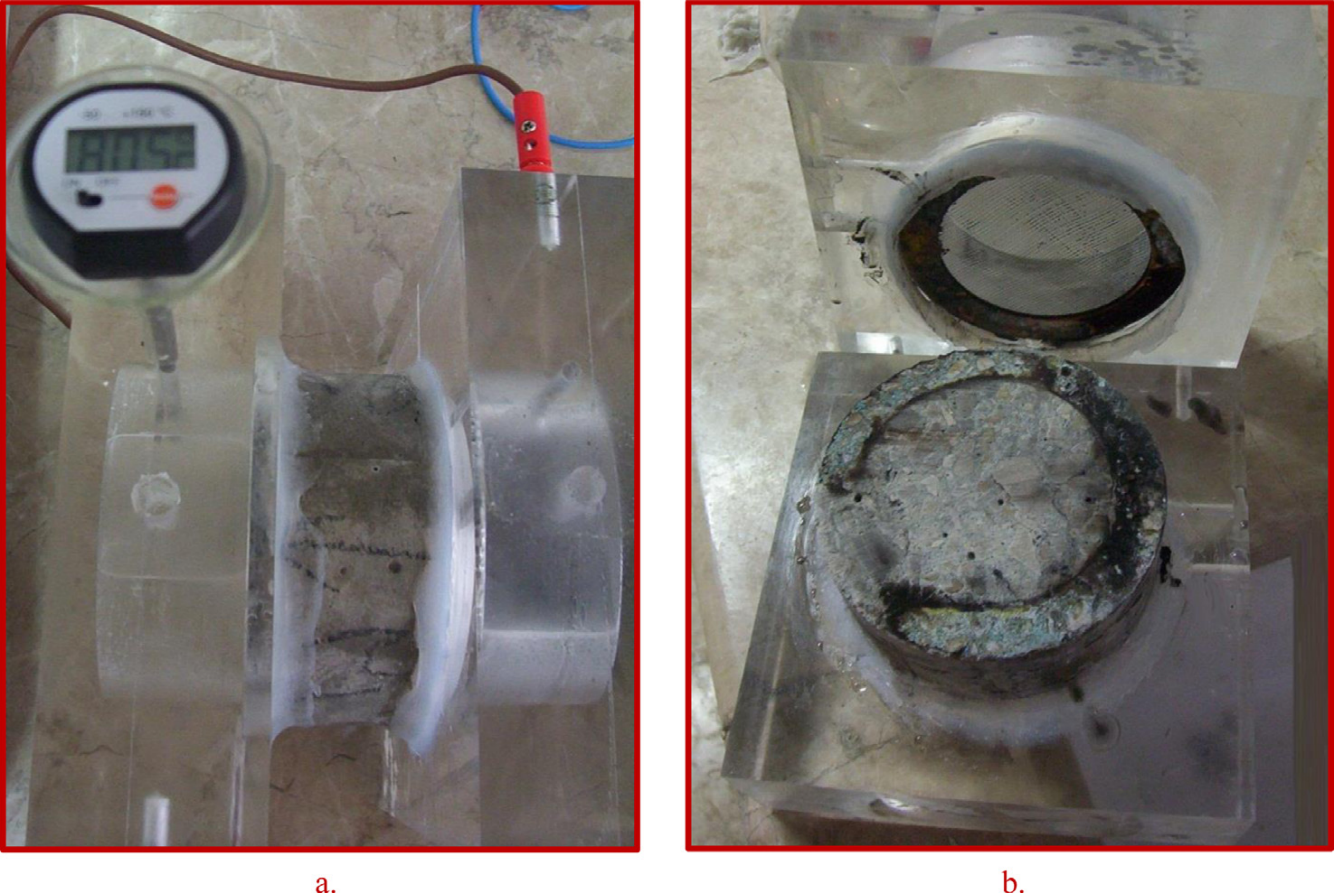
Photograph of a control concrete slice of w/b=0.7 after CIP test. The test was stopped after about 5 hours. The temperature inside the cell raised up to more than 80 °C (a), because of the highly penetrable concrete specimen (b).

**Fig. 4:**
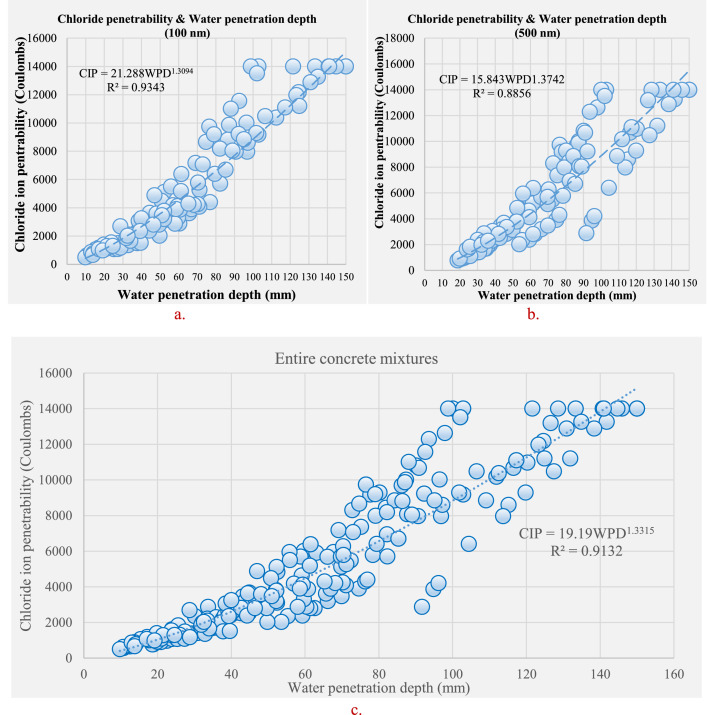
relationship between WPD and CIP for the concrete mixtures containing NNP of 100 nm size (a), the concrete mixtures containing NNP of 500 nm size (b) and the entire concrete mixtures (c).

Table 1 displays the chemical composition of the cement, natural pozzolana and the coarse aggregates. Figs. 1 & 2 display the surfaces of chloride ion penetrability values for the concretes prepared using nano natural pozzolana of either 100 or 500 nm size. Fig. 3 shows a highly penetrable concrete specimen prepared with a w/b of 0.7. An attempt was made to correlate water permeability with chloride ion penetrability as shown in Fig. 4. More data can be found in the supplementary file (https://data.mendeley.com/datasets/m9tsgpvrtm/1), which is of an Excel format of five sheets. The first two sheets nominated “WPD-all results” and “CIP-all results” contain the entire results of WPD and CIP tests, respectively, along with the computed average and standard deviation values. The other two sheets nominated “Results of 100 nm-based concrete” & “Results of 500 nm-based concrete” contain the average values of WPD and CIP tests along with some representative figures for concrete specimens prepared with nano natural pozzolana of 100 & 500 nm size, respectively. The fifth sheet nominated “Correlation between WPD & CIP” was constructed to establish a correlation between WPD and CIP. Some further data can be also obtained from Refs. [1], [2], [3].

**Table 1:** Chemical composition of the ingredients in concrete mixtures

	Concrete ingredients			
Main oxides (%)	Natural Pozzolana	Portland Cement	Coarse aggregates	Natural sand
SiO_2_	45.4	19.6	0.4	93.4
CaO	9.3	62.3	31.4	1.7
Al_2_O_3_	15.7	4.8	0.4	0.6
Fe_2_O_3_	10.4	3.6	0.1	0.3
MgO	8.2	2.8	20.5	0.2
SO_3_	0.3	2.1	0.2	1.2
Alkalis (Na_2_O + K_2_O)	3.9	0.6	0.4	0.1
Cl^−^	<<0.1	<<0.1	<<0.1	<<0.1
Loss On Ignition (LOI)	0.6	1.4	46.5	2.5

## 2 Experimental Design, Materials and Methods

The data presented in the current paper are results of experimental tests conducted by the author. Concrete mixtures were prepared with six replacement levels of NNP; namely 0%, 1%, 2%, 3%, 4% and 5%, four water/binder ratios, namely 0.4, 0.5, 0.6 and 0.7. NP was ground to 2 different sizes; i.e. 100 & 500 nm.

Natural pozzolana (NP) was quarried from a region situated at the northeast of Harrat al-Shaam, which is a basaltic volcanic field of about fifty thousand km^2^ spreading over Syrian, Jordanian and Saudi territories. The chemical composition of NP is presented in Table 1. The main minerals traced in NP are Anorthite, Forstrite, Fujasite, Diopside and Calcite. NP because of its vesicular nature has a bulk density value of less than 0.7 [3]. To reach the studied sizes; namely 100 nm & 500 nm, NP was ground for 360 min and 275 min, respectively, using a laboratory centrifugal ball mill (Retsch, S100, Germany). The adopted NP dispatch/steel ball ratio was 1/5 at a revolution number of 300 rpm [2].

The cement used in concrete mixtures was obtained from Adra Plant “a local cement plant located in Damascus’ northeastern countryside”. It was ground to a Blaine fineness value of 360 m^2^/kg with a median particle size of about 15 µm. Its chemical composition is illustated in Table 1. The mineralogical composition computed based on the Bogue formuals is: C_3_S (61%), C_2_S (10%), C_3_A (7%) and C_4_AF (11%). Its setting times: 162 min (initial) and 224 min (final).

Crushed dolomitic aggregates with three sizes were used in making concrete mixtures. The first size was crushed fine aggregate of a nominal max size of 4.75 mm (Relative density=2.8; absorption (A) (%)=1.57) while the second and third sizes are crushed medium-size aggregate (Relative density=2.83; A (%)=1.30; Los Angeles (LA) Number=20.5) and crushed coarse aggregate (Relative density=2.83; A (%)=1.30; LA Number=20.5) of nominal max sizes of 12.5 and 25 mm, respectively. Natural silicious river sand of a nominal max size of 1.18 mm (Relative density=2.69; A (%)=1.60) was incorporated in order to bring the aggregate blend’ grading as close as possible to the Fuller's grading. Water used in the preparation of all concrete mixtures is a drinkable water.

ACI 211 guidelines were adopted in designing the investigated concrete mixtures. Slump values of 150 ± 25 mm were kept in all concrete mixtures. In addition, the percentage of coarse aggregate in the aggregates blend was also kept constant. 0, 5, 2 and 4 l/m^3^ of superplasticizer “type F” (ASTM C494) were added to the fresh concrete mixtures of w/b ratios of 0.7, 0.6, 0.5 and 0.4, respectively. The binder content was fixed in all mixtures; i.e. 350 kg binder per one cubic meter. The detailed mixing process can be found in ref. nr. [3].

After five curing ages, i.e. 2, 7, 28, 90 and 180 days, all hardened concrete specimens were tested for either water permeability or chloride ion penetrability. The international standards, i.e. EN 12390-8 & ASTM C1202 were adopted to test the water permeability of concrete cubic specimens of 150 mm and the chloride ion penetrability of concrete cylindrical slices of (100 mm × 50 mm), respectively.

**Fig. 5:**
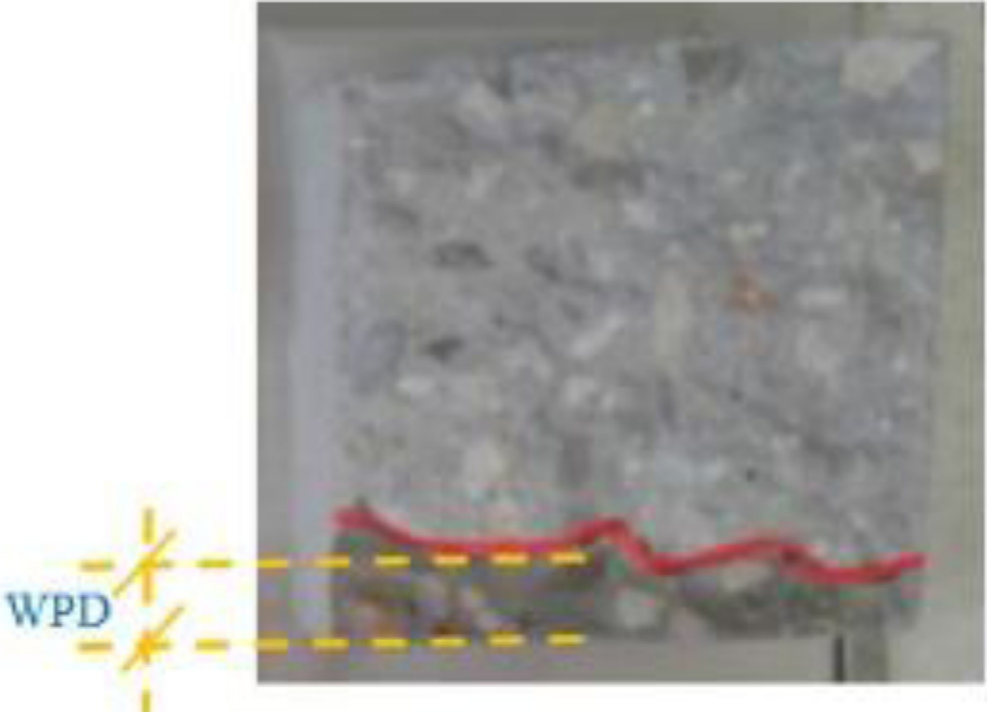
Photograph of a broken tested concrete specimen prepared using 3% NNP of 500 nm size and cured for 28 days. The measurement of WPD conducted on the specimen indicates 26.2 mm on average.

In the concrete water permeability test, water from the bottom surface was forced to penetrate under a 5 bar-constant presser applied for 72 hours. The concrete specimen was then broken in order to determine the water penetration depth (WPD), Fig. 5 . WPD i.e. (high and low) was recorded for each tested specimen [4]. On the other hand, the chloride ion penetrability test was conducted on slices of 100 mm in diameter and 50 mm in thickness cut from the middle part of each concrete cylinder specimens (100 mm × 200 mm). The total charge, in coulombs, passed through the concrete slice, was determined at the end of the test, which takes a period of 6 hours. Concrete specimen of total charge value ≤ 2000 coulombs can be classified as low permeable concrete (ASTM C1202).

## Ethics Statements

Not applicable.

## CRediT Author Statement

The author is the sole responsible for the paper.

## Declaration of competing interest

The author declares that he has no known competing financial interests or personal relationships that could have appeared to influence the work reported in this paper.

## Data Availability

Datainbrief-21.05.2022-all results-revised (Original data) (Mendeley Data).
